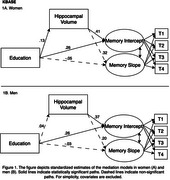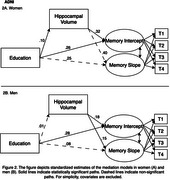# Sex/gender differences in neurodegeneration pathways linking education to cognitive trajectories among older adults in Korean and US research study cohorts

**DOI:** 10.1002/alz70860_106933

**Published:** 2025-12-23

**Authors:** Diefei Chen, C. Elizabeth Shaaban, Kacie D Deters, Jeremy A. Tanner, Talia L. Robinson, Adam M. Staffaroni, Ileana De Anda‐Duran, Stefanie Pina‐Escudero, Martine Elbejjani, Paul K Crane, Shannon L Risacher, Dahyun Yi, Min Soo Byun, Dong Young Lee, Kwangsik Nho, Andrew J. Saykin, Meichen Yu, Evgeny J. Chumin, A. Zarina Kraal

**Affiliations:** ^1^ Department of Epidemiology, Bloomberg School of Public Health, Johns Hopkins University, Baltimore, MD, USA; ^2^ University of Pittsburgh, Pittsburgh, PA, USA; ^3^ University of Pittsburgh Alzheimer's Disease Research Center (ADRC), Pittsburgh, PA, USA; ^4^ University of California, Los Angeles Integrative Biology and Physiology (IBP), Los Angeles, CA, USA; ^5^ University of Texas Health San Antonio, San Antonio, TX, USA; ^6^ Brigham and Women's Hospital, Boston, MA, USA; ^7^ Massachusetts General Hospital, Boston, MA, USA; ^8^ University of California, San Francisco, San Francisco, CA, USA; ^9^ Memory and Aging Center, UCSF Weill Institute for Neurosciences, University of California, San Francisco, San Francisco, CA, USA; ^10^ UCSF Alzheimer's Disease Research Center, San Francisco, CA, USA; ^11^ Memory and Aging Center, Weill Institute for Neurosciences, University of California, San Francisco, San Francisco, CA, USA; ^12^ Celia Scott Weatherhead Tulane University School of Public Health and Tropical Medicine, New Orleans, LA, USA; ^13^ University of Califormia San Francisco, San Francisco, CA, USA; ^14^ American University of Beirut, Beirut, Beirut, Lebanon; ^15^ University of Washington School of Medicine, Seattle, WA, USA; ^16^ Indiana Alzheimer's Disease Research Center, Indiana University School of Medicine, Indianapolis, IN, USA; ^17^ Seoul National University Medical Research Center, Seoul, Korea, Republic of (South); ^18^ Department of Radiology and Imaging Sciences, Indiana Alzheimer's Disease Research Center, Center for Neuroimaging, Indiana University School of Medicine, Indianapolis, IN, USA; ^19^ School of Medicine, Indiana University, Indianapolis, IN, USA; ^20^ Indiana Alzheimer's Disease Research Center, Indiana University School of Medicine, Indianapolis, IN, USA; ^21^ Taub Institute for Research on Alzheimer's Disease and the Aging Brain, New York, NY, USA

## Abstract

**Background:**

Educational attainment protects against poor cognitive health outcomes in later‐life via beneficial impacts on brain health in regions implicated in Alzheimer's disease (AD). However, brain health mechanisms underlying the protective effect of education on cognition may differ between men and women and across different racialized and ethnic groups, given differences in their educational opportunities. This study aims to characterize sex/gender differences in the effects of education on later‐life cognitive trajectories in Korean and US research study cohorts.

**Method:**

Data were from older adults without dementia in the Korean Brain Aging Study for the Early Diagnosis and Prediction of Alzheimer's Disease (KBASE: *N* = 434, age=70±8 years, education = 11±5 years, 57% women) and the Alzheimer's Disease Neuroimaging Initiative (ADNI3: *N* = 375, age=71±7 years, education = 17±2 years, 53% women; all non‐Latinx white). Latent growth curves examined whether there were sex/gender differences in the mediating roles of baseline hippocampal volume and cortical thickness AD‐signature regions (separately) on the associations between education and four‐year memory trajectories. Models were stratified by sex/gender, cohort, adjusting for age, *APOE* genotype, and cardiometabolic conditions (in KBASE).

**Result:**

All mediation models fit well. Among women in KBASE, hippocampal volume partially mediated the association between greater educational attainment and higher memory level (indirect effect: β=.06, SE=.02, *p* = .02) and slower memory decline (indirect effect: β=.04, SE=.02, *p* = .04; Figure 1). Among women in ADNI, a similar pattern of association was observed for memory level (indirect effect: β=.04, SE=.02, *p* = .04; Figure 2) but not memory change. Mediation was not observed in men in KBASE and ADNI. Cortical thickness did not mediate the effects of education on memory in either cohort.

**Conclusion:**

Our findings highlight potential sex/gender differences in neurobiological pathways linking education to cognition, while differences in effects on memory decline point to differences in mechanisms underlying memory trajectories across cohorts. Future research is needed confirm causal effects and characterize sociocultural factors that may shape educational opportunities and brain health across diverse populations.